# miRNome Reveals New Insights Into the Molecular Biology of Field Cancerization in Gastric Cancer

**DOI:** 10.3389/fgene.2019.00592

**Published:** 2019-06-19

**Authors:** Adenilson Pereira, Fabiano Moreira, Tatiana Vinasco-Sandoval, Adenard Cunha, Amanda Vidal, André M. Ribeiro-dos-Santos, Pablo Pinto, Leandro Magalhães, Mônica Assumpção, Samia Demachki, Sidney Santos, Paulo Assumpção, Ândrea Ribeiro-dos-Santos

**Affiliations:** ^1^Laboratory of Human and Medical Genetics, Institute of Biological Sciences, Graduate Program of Genetics and Molecular Biology, Federal University of Pará, Belém, Brazil; ^2^Research Center on Oncology, Graduate Program of Oncology and Medical Science, Federal University of Pará, Belém, Brazil

**Keywords:** miRNome, miRNAs, gastric cancer, field cancerization, biomarkers

## Abstract

**Background:**

MicroRNAs (miRNAs) play an important role in gastric carcinogenesis and have been associated with gastric field cancerization; however, their role is not fully understood in this process. We performed the miRNome sequencing of non-cancerous, adjacent to tumor and gastric cancer samples to understand the involvement of these small RNAs in gastric field cancerization.

**Methods:**

We analyzed samples of patients without cancer as control (non-cancerous gastric samples) and adjacent to cancer and gastric cancer paired samples, and considered miRNAs with |log_2_(*fold change*)| > 2 and *Padj* < 0.05 to be statistically significant. The identification of target genes, functional analysis and enrichment in KEGG pathways were realized in the TargetCompare, miRTargetLink, and DAVID tools. We also performed receiver operating characteristic (ROC) curves and miRNAs that had an AUC > 0.85 were considered to be potential biomarkers.

**Results:**

We found 14 miRNAs exclusively deregulated in gastric cancer, of which six have potential diagnostic value for advanced disease. Nine miRNAs with known tumor suppressor activities (TS-miRs) were deregulated exclusively in adjacent tissue. Of these, five have potential diagnostic value for the early stages of gastric cancer. Functional analysis of these TS-miRs revealed that they regulate important cellular signaling pathways (PI3K-Akt, HIF-1, Ras, Rap1, ErbB, and MAPK signaling pathways), that are involved in gastric carcinogenesis. Seven miRNAs were differentially expressed in both gastric cancer and adjacent regarding to non-cancerous tissues; among them, *hsa-miR-200a-3p* and *hsa-miR-873-5p* have potential diagnostic value for early and advanced stages of the disease. Only *hsa-miR-196a-5p* was differentially expressed between adjacent to cancer and gastric cancer tissues. In addition, the other miRNAs identified in this study were not differentially expressed between adjacent to cancer and gastric cancer, suggesting that these tissues are very similar and that share these molecular changes.

**Conclusion:**

Our results show that gastric cancer and adjacent tissues have a similar miRNA expression profile, indicating that studied miRNAs are intimately associated with field cancerization in gastric cancer. The overexpression of TS-miRs in adjacent tissues may be a barrier against tumorigenesis within these pre-cancerous conditions prior to the eventual formation or relapse of a tumor. Additionally, these miRNAs have a great accuracy in discriminating non-cancerous from adjacent to tumor and cancer tissues and can be potentially useful as biomarkers for gastric cancer.

## Introduction

Gastric cancer (GC) is an aggressive disease that is considered the third leading cause of cancer death worldwide (Ferlay et al., 2015). GC is usually diagnosed in late stages, due in part to the limited efficiency of existing biomarkers, and the 5-year survival rates of these patients do not exceed 30% (Correa, 2013; Yakirevich and Resnick, 2013). Studies have shown that microRNAs (miRNAs) have excellent sensitivity/specificity and a high discriminatory capacity, which make them potentially useful as molecular biomarkers for this type of cancer (Wu et al., 2014; Shin et al., 2015; Vidal et al., 2016).

MicroRNAs are small non-coding RNAs (∼18–30 nt) that regulate gene expression post-transcriptionally, disrupting the expression of target mRNAs (Bartel, 2004; Wu et al., 2014). These molecules play an important role in multiple pathways and the processes responsible for the maintenance of healthy tissue homeostasis (Schneider, 2012; Runtsch et al., 2014). In humans, studies suggest that miRNAs are part of the complex regulatory network of the healthy stomach (Ribeiro-dos-Santos et al., 2010; Moreira et al., 2014a). Therefore, when miRNAs are deregulated in the human stomach, they compromise important pathways that regulate the normal functions of this organ (Zhang et al., 2014), contributing to the onset and progression of gastric carcinogenesis (Wu et al., 2014).

In recent years, our research group joined efforts and used different technologies to understand the relationship between the deregulation of these small RNAs and gastric carcinogenesis (Assumpção et al., 2015; Darnet et al., 2015; Vidal et al., 2016; Magalhães et al., 2018; Pereira et al., 2019). More recently, we have demonstrated the deregulation of miRNAs during the evolution of Correa’s cascade (Vidal et al., 2016) and in field cancerization in GC (Assumpção et al., 2015; Pereira et al., 2019).

Field cancerization assumes that the tissues adjacent to the tumor have molecular changes (e.g., genetic and/or epigenetic) that make them susceptible to the onset of tumors or recurrences (Slaughter et al., 1953; Chai and Brown, 2009; Curtius et al., 2018). The preexisting molecular changes in this tissue (which may be caused by an insult to the healthy epithelium) are precursors of carcinogenesis and serve as background for the establishment and progression of the tumor (Hattori and Ushijima, 2016; Padmanabhan et al., 2017; Curtius et al., 2018). In fact, studies have shown that adjacent to the tumor tissues have similar molecular changes to those found in the tumor as well as unique and exclusive changes, which distinguish them from non-cancerous tissues (Assumpção et al., 2015; Takeshima et al., 2015; Aran et al., 2017; Vidal et al., 2017; Yoshida et al., 2017; Pereira et al., 2019). Recently, miRNAs were related to field cancerization in GC (Assumpção et al., 2015; Pereira et al., 2019), however, little is known about the true role played by these small non-coding RNAs in this process. The use of robust large-scale sequencing technologies is an excellent strategy in both the discovery of new biomarkers and in providing an overview of the complex relationship between miRNAs and field cancerization.

In this study, we used deep sequencing to evaluate the overall expression profile of miRNAs in non-cancerous gastric tissues, adjacent to cancer and with cancer tissues in order to identify miRNAs involved in the field cancerization. In addition, we evaluated the discriminatory performance of miRNAs as biomarkers of gastric carcinogenesis. Our results show new deregulated miRNAs, which are potentially useful as biomarkers for this cancer and suggest a new molecular mechanism involved in the biology of field cancerization.

## Materials and Methods

### Biological Material

A total of 45 fresh samples of stomach antrum tissues were included in the present study. The non-cancerous control (NC) samples were collected from 15 patients without cancer (chronic gastritis; ± *H. pylori*; mean age = 59.2) during an upper digestive endoscopy (tissue fragments of approximately 4 millimeters). In addition, 15 tumor-adjacent tissues (histopathologically without cancer) and 15 gastric adenocarcinoma samples were collected from patients with gastric cancer. The tumor-adjacent (ADJ) and gastric adenocarcinoma (GC) samples were all paired (± *H. pylori*; mean age = 59.9).

The tissues were obtained from patients treated at the Hospital Universitário João de Barros Barreto (HUJBB), Belém, Pará, Brazil. Samples were collected prior to antibiotic, chemotherapeutic, and/or radiotherapeutic treatment. Immediately after collection, all samples were frozen and stored in liquid nitrogen until analysis.

Histopathological characterization of the samples, such as tumor subtype, degree of differentiation, depth of invasion, involvement of lymph nodes, and/or distant metastases were extracted from pathological reports performed by the HUJBB Department of Pathology. Histopathological analysis of the tumor fragments was performed according to Lauren’s classification (Lauren, 1965).

### Ethics Statement

This study was reviewed and approved by the Ethics Committee of the Center of Oncology Research of the Federal University of Pará (Protocol No. 1.081.340). All study participants or their legal guardian provided informed written consent in accordance with the Helsinki Declaration of 1964, the Nuremberg Code, in compliance with the National Health Council’s Research Guidelines Involving Human Beings (Res. CNS 466/12).

### RNA Extraction, Small RNA Library Construction, and Sequencing

Total RNA was extracted using TRIzol^®^ reagent (Thermo Fisher Scientific). After isolation, total RNA was stored at -80°C until further analysis. The total RNA amount was determined using a Qubit^®^2.0 (Life Technologies, Foster City, CA, United States), and an Agilent RNA ScreenTape assay and 2200 TapeStation Instrument (Agilent Technologies, United States) were used to detect RNA integrity. Samples with an RNA integrity number (RIN) ≥ 5 were sequenced.

For small RNA-seq, 1 μg of total RNA per sample was used for library preparation utilizing TruSeq Small RNA Sample Prep Kits (Illumina, San Diego, CA, United States). A DNA ScreenTape assay in a 2200 TapeStation Instrument (Agilent Technologies, United States) and real-time PCR with a KAPA Library Quantification Kit (KAPABIOSYSTEM, United States) were used to validate and quantify each library. A 4-nM library pool comprising all samples was sequenced using a MiSeq reagent kit v3 150 cycle on a MiSeq System (Illumina, San Diego, CA, United States).

The raw sequencing reads of all libraries have been deposited at EBI-ENA (PRJEB27213).

### Bioinformatics Analysis

The resulting reads were pre-processed and quality filtered (qv > 25). We used STAR (Dobin et al., 2013) aligner to map the reads to the human genome reference (GRCH37). We quantified mature miRNA sequencing using miRBase human annotation (v20). Counting expression data was performed with HTSeq (Anders et al., 2015).

Differential expression analysis of all processed data was performed using the bioconductor-DESeq2 package (Love et al., 2014) in R software, with a detection threshold of 10 counts per miRNA (present at least 10 read counts in at least of the libraries). Comparison between (i) gastric cancer (GC) vs. non-cancerous (NC) samples; (ii) adjacent to gastric cancer (ADJ) vs. NC samples; and (iii) GC vs. ADJ samples were made separately. Adjusted *P*-values ≤ 0.05 and |log_2_(*fold change*)| > 2 were considered statistically significant.

For graphical analysis of miRNAs, expression data was normalized to RPKM. Heatmaps were used for hierarchical clustering of differentially expressed miRNAs. The area under the curve (AUC > 0.85) from the receiver operating characteristic (ROC) curves was used to identify biomarkers with the best sensitivity/specificity relation, and a discriminant analysis of principal components (DAPC) was constructed to infer the number of clusters of epigenetically related samples. All graphical analyses were performed using the R statistical platform.

### Identification of Differentially Expressed miRNA Target Genes

We used two online tools to identify the differentially expressed (D. E.) miRNA target genes: (i) TargetCompare (Moreira et al., 2014b) and (ii) miRTargetLink Human (Hamberg et al., 2016). TargetCompare database allows the user to filter miRNAs and its targets genes that are associated with determined diseases, such as GC. miRTargetLink Human is a tool that allows the search for interactions between target genes and miRNAs that have been experimentally validated by molecular biology techniques.

The identified target genes were submitted to functional annotation and enrichment in KEGG pathways using DAVID Bioinformatics Resources v.6.8 online tool (Huang et al., 2009a, 2009b).

## Results

After quality control (Supplementary Figure S1), alignment and transcript quantitation, several small non-coding RNAs (sncRNAs) and other transcript fragments were identified. From them, ∼30% (∼9.5 million reads) were recognized as microRNAs reads, identifying 1,144 mature miRNAs. Approximately 90% of the miRNA reads (∼8.5 million reads) were concentrated on the 35 most expressed miRNAs. The number and representativity of D. E. expressed miRNAs identified in this study is similar to other studies that performed miRNome analysis (Hou et al., 2011; Maltseva et al., 2014; Liu et al., 2015; Castro-Magdonel et al., 2017; Liang et al., 2017).

### Differentially Expressed miRNAs in GC vs. NC Analysis

**FIGURE 1:**
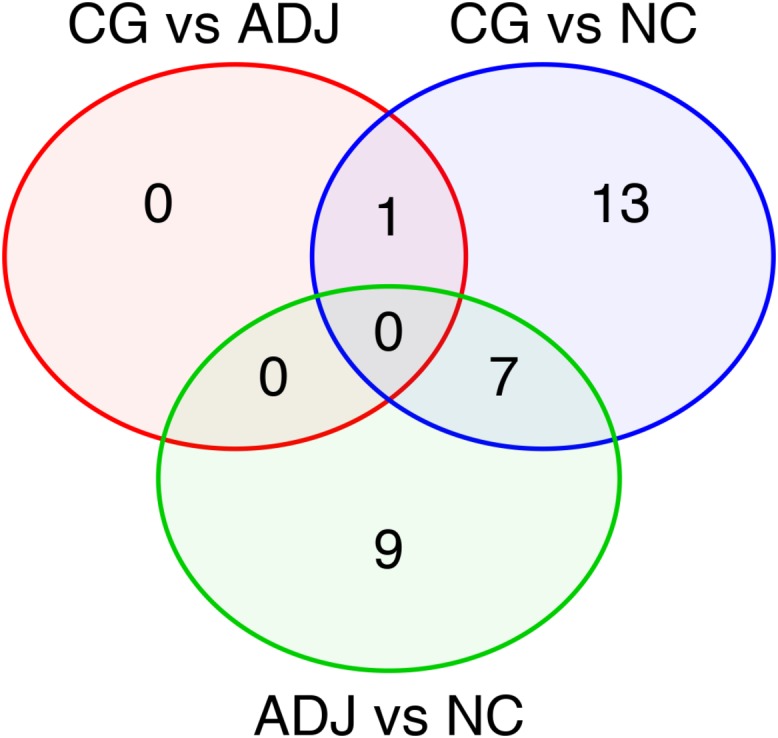
Differentially expressed miRNAs in all comparisons made among the studied groups. Venn’s Diagram of the differentially expressed miRNAs shared between GC vs. ADJ, GC vs. NC, and ADJ vs. NC analysis.

**FIGURE 2:**
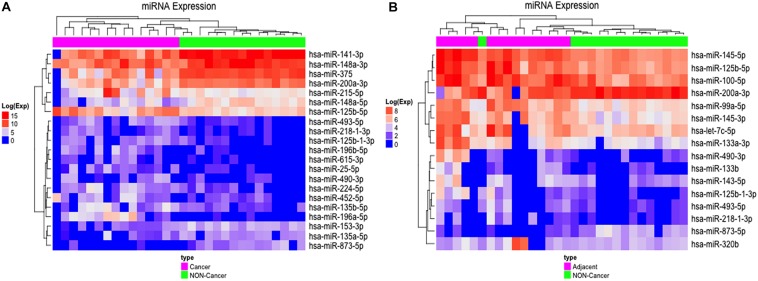
Differentially expressed miRNAs in GC vs. NC and ADJ vs. NC analysis. **(A)** Heatmap of the 21 most highly expressed miRNAs in each of the two distinct groups: GC (in purple) and NC (in green). **(B)** Heatmap of the 16 most highly expressed miRNAs in each of the two groups: ADJ (in purple) and NC (in green) samples.

We found 21 differentially expressed (D. E.) miRNAs when we compared gastric cancer samples (GC) with non-cancerous gastric samples (NC) (Figure 1 and Supplementary Table S1), of which eight were down-regulated and 13 were up-regulated in GC (Supplementary Table S1). The heatmap obtained from the normalized expression of the D. E. miRNAs perfectly clustered the GC and NC samples together (Figure 2A).

**FIGURE 3:**
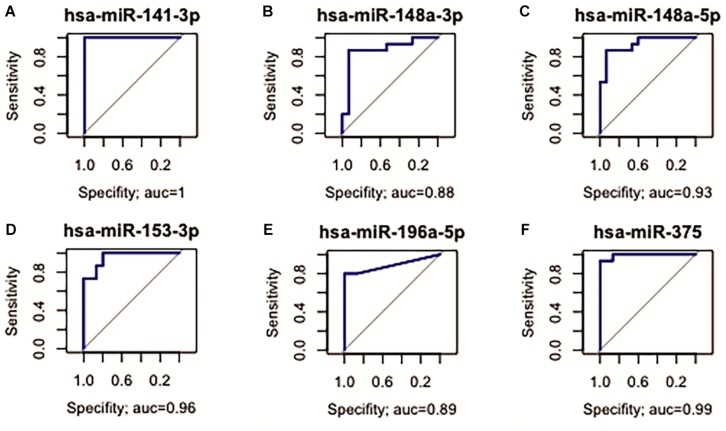
Receiver operating characteristic (ROC) curve analysis identified miRNAs with potential to identify GC. **(A)** *hsa-miR-141-3p* (AUC = 1; 95% CI: 1.000–1.000^*^); **(B)** *hsa-miR-148a-3p* (AUC = 0.88; 95% CI: 0.733–1.000); **(C)** *hsa-miR-148a-5p* (AUC = 0.93; 95% CI: 0.840–1.000); **(D)** *hsa-miR-153-3p* (AUC = 0.96; 95% CI: 0.893–1.000); **(E)** *hsa-miR-196a-5p* (AUC = 0.89; 95% CI: 0.767–1.000); **(F)** *hsa-miR-375* (AUC = 0.99; 95% CI: 0.970–1.000).

Fourteen miRNAs were exclusively D. E. in the GC vs. NC analysis: nine were up-regulated (six oncomiRs, one TS-miR and two with unknown functions in GC) and five were down-regulated (all TS-miRs) in GC (Figure 1 and Table 1). Six miRNAs, of which five were down-regulated (*hsa-miR-141-3p*, *-miR-148a-3p*, *-miR-148a-5p*, *-miR-153-3p*, and *-miR-375*) and one were up-regulated (*hsa-miR-196a-5p*), presented the best sensitivity/specificity relation (AUC > 0.85) and were considered as potential biomarkers to identify GC (Figure 3).

**TABLE 1:** Deregulated miRNAs only in gastric cancer.

**miRNA**	Role in GC^*^	**GC vs. NC**	**ADJ vs. NC**	**GC vs. ADJ**
*miR-135b-5p*	OncomiR^1,2,3^	Up	–	–
*miR-196a-5p*	OncomiR^4,5^	Up	–	Up
*miR-196b-5p*	OncomiR^6^	Up	–	–
*miR-215-5p*	OncomiR^7^	Up	–	–
*miR-224-5p*	OncomiR^8^	Up	–	–
*miR-615-3p*	OncomiR^9^	Up	–	–
*miR-25-5p*	OncomiR^10,11^	Up	–	–
*miR-141-3p*	TS-miR^12,13^	Up	–	–
*miR-452-5p*	TS-miR^14^	Up	–	–
*miR-135a-5p*	TS-miR^15,16^	Down	–	–
*miR-148a-3p*	TS-miR^17^	Down	–	–
*miR-148a-5p*	TS-miR^17^	Down	–	–
*miR-153-3p*	TS-miR^18,19^	Down	–	–
*miR-375*	TS-miR^20,21,22^	Down	–	–

GC, gastric cancer; ADJ, adjacent to cancer; NC, non-cancerous; Up, up-regulated; Down, down-regulated; TS-miR, tumor suppressor miRNA; OncomiR, oncogenic miRNA. (-) Not differentially expressed. (^*^) Putative role in GC. (1) Wang et al., 2012; (2) Lu et al., 2018; (3) Shao et al., 2019; (4) Sun et al., 2012; (5) Pan et al., 2017; (6) Liao et al., 2012; (7) Deng et al., 2014; (8) He et al., 2017; (9) Wang et al., 2018; (10) Li B.S. et al., 2015; (11) LArki et al., 2018; (12) Zuo et al., 2015; (13) Zhou et al., 2019; (14) Gao et al., 2016; (15) Zhang et al., 2016; (16) Xie et al., 2019; (17) Zheng et al., 2011; (18) Wang and Liu, 2016; (19) Ouyang et al., 2018; (20) Ding et al., 2010; (21) Chen et al., 2017; (22) Hwang et al., 2018.

### Differentially Expressed miRNAs in ADJ vs. NC Analysis

Comparing adjacent to gastric cancer samples (ADJ) with NC revealed 16 D. E. miRNAs (Figure 1 and Supplementary Table S2), of which two were down-regulated and 14 were up-regulated (Supplementary Table S2). The heatmap obtained from the normalized expression of the D. E. miRNAs clustered ADJ and NC samples together (Figure 2B). Furthermore, nine miRNAs (eight TS-miRs and one with unknown function in GC) were exclusively D. E. in the ADJ vs. NC analysis: they were all up-regulated in ADJ (Figure 1 and Table 2).

**TABLE 2:** Deregulated miRNAs only in adjacent to tumor tissue.

**miRNA**	Role in GC^*^	**GC vs. NC**	**ADJ vs. NC**	**GC vs. ADJ**
*miR-let7-c-5p*	TS-miR^1^	–	Up	–
*miR-133a-3p*	TS-miR^2,3^	–	Up	–
*miR-133b*	TS-miR^2,4^	–	Up	–
*miR-143-5p*	TS-miR^5,6^	–	Up	–
*miR-145-3p*	TS-miR^5,6^	–	Up	–
*miR-145-5p*	TS-miR^5,6^	–	Up	–
*miR-99a-5p*	?	–	Up	–
*miR-100-5p*	TS-miR^7^	–	Up	–
*miR-320b*	TS-miR^8^	–	Up	–

GC, gastric cancer; ADJ, adjacent to cancer; NC, non-cancerous; Up, up-regulated; TS-miR, tumor suppressor miRNA; OncomiR, oncogenic miRNA. (-) Not differentially expressed. (^*^) Putative role in GC. (1) Tsai et al., 2015; (2) Qiu et al., 2014; (3) Zhang et al., 2018; (4) Yang et al., 2017; (5) Wu et al., 2013; (6) Lei et al., 2017; (7) Shi et al., 2015; (8) Zhao et al., 2017.

**FIGURE 4:**
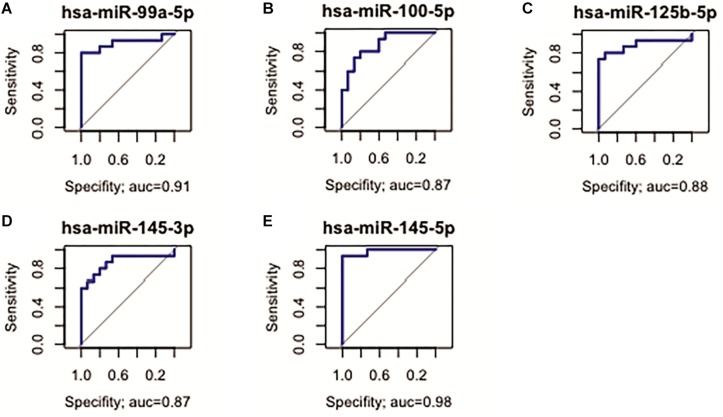
Receiver operating characteristic curve analysis of the five selected miRNAs with potential to predict alterations in ADJ tissue. **(A)** *hsa-miR-99a-5p* (AUC = 0.91; 95% CI: 0.783–1.000); **(B)** *hsa-miR-100-5p* (AUC = 0.87, 95% CI: 0.747–0.995); **(C)** *hsa-miR-125b-5p* (AUC = 0.88; 95% CI: 0.742–1.000); **(D)** *hsa-miR-145-3p* (AUC = 0.87; 95% CI: 0.721–1.000); **(E)** *hsa-miR-145-5p* (AUC = 0.98; 95% CI: 0.944–1.000).

Five D. E. miRNAs (*hsa-miR-99a*, *-miR-100-5p*, *-miR-125b-5p*, *-miR-145-3p*, and *-miR-145b-5p*, all up-regulated in ADJ) presented the best sensitivity/specificity relation (AUC > 0.85) and were considered as potential early biomarkers for GC (Figure 4).

### Functional Analysis of the Up-Regulated TS-miRs in ADJ Tissue

**FIGURE 5:**
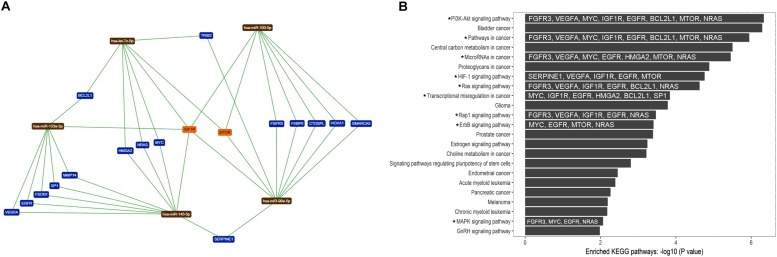
Target genes common to the five up-regulated TS-miRs in adjacent tissue and their relationship to important biological pathways. **(A)** Network of interaction among miRNAs and target genes generated by miRTargetLink Human (Hamberg et al., 2016). **(B)** Enhanced KEGG biological pathways in which the target genes of the studied miRNAs participate (by DAVID v.6.8). (^*^) Important pathway in GC. The target genes involved in important pathways in GC are in white inside the dark bars.

To evaluate the biological and functional role of the eight up-regulated TS-miRs and *hsa-miR-99a-5p* in ADJ tissue (Table 2), we performed enrichment and functional annotation of their experimentally validated common target genes. We used nine miRNAs as input in the online tools TargetCompare (Moreira et al., 2014b) and miRTargetLink Human (Hamberg et al., 2016), which output five miRNAs (*hsa-miR-let7-c-5p*, *-miR-99a-5p*, *-miR-100-5p*, *-miR-133a-3p*, and *-miR-145-5p*) that regulate 18 common target genes (Figure 5A). The enrichment and functional annotation analysis of the 18 target genes performed in DAVID v.6.8 (Huang et al., 2009a, 2009b) revealed that 13 genes participate in 23 different biological pathways (Supplementary Table S3), and 11 genes are involved in nine biological pathways that are important for the development and progression of GC (Figure 5B).

### Differentially Expressed miRNAs Common to GC vs. ADJ vs. NC Analysis

**FIGURE 6:**
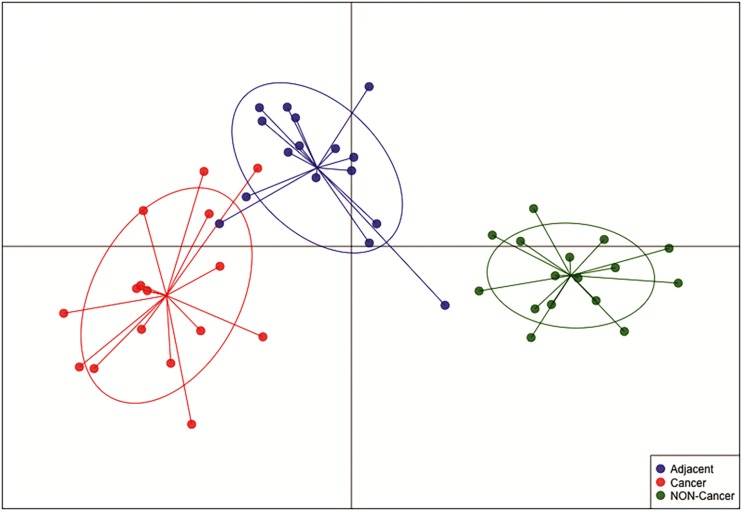
Discriminant analysis of principal component (DAPC) plot shows that NC, ADJ, and GC samples form distinct clusters. The DAPC analysis showed great similarity between GC and ADJ tissues.

**FIGURE 7:**
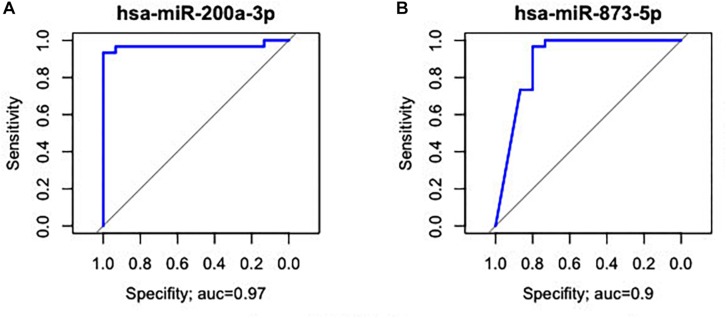
Receiver operating characteristic curve analysis of the two miRNAs with the potential to predict changes in both ADJ and GC tissues. **(A)** *hsa-miR-200a-3p* (AUC = 0.97; 95% CI: 0.983–1.000). **(B)** *hsa-miR-873-5p* (AUC = 0.90; 95% CI: 0.807–1.000).

*hsa-miR-125b-5p*, *miR-125b-1-3p*, *-miR-200a-3p*, *miR-218-1-3p*, *-miR-490-3p*, *-miR-493-5p* and *-miR-873-5p* were D. E. in both the GC vs. NC (Table 3 and Supplementary Table S1) and ADJ vs. NC (Table 3 and Supplementary Table S2) analyses. These miRNAs were not D. E. in the GC vs. ADJ analysis (Table 3), suggesting that these two tissues are similar regarding the expression of these miRNAs. In addition, the DAPC plot (Figure 6) generated by all D. E. miRNAs indicates that GC and ADJ, despite generating distinct clusters, have much more similarity in its expression profiles when compared with NC tissue, which clustered apart. Thus, we assembled the GC and ADJ samples in a single group to compare to the NC samples during the ROC curve analysis. Two down-regulated miRNAs (*hsa-miR-200a-3p* and *hsa-miR-873-5p*) had the best sensitivity/specificity relation and were considered as potential biomarkers to identify gastric carcinogenesis (Figures 7A,B).

**TABLE 3:** Deregulated miRNAs considering both adjacent to tumor and gastric cancer tissues.

**miRNA**	Role in GC^*^	**GC vs. NC**	**ADJ vs. NC**	**GC vs. ADJ**
*miR-125b-5p*	OncomiR^1,2^	Up	Up	–
*miR-125b-1-3p*	OncomiR^1,2^	Up	Up	-
*miR-218-1-3p*	TS-miR^3,4,5,6^	Up	Up	-
*miR-490-3p*	TS-miR^7,8,9^	Up	Up	–
*miR-200a-3p*	TS-miR^10^	Down	Down	–
*miR-873-5p*	TS-miR^11,12^	Down	Down	–
*miR-493-5p*	TS-miR^13^	Down	Up	–

GC, gastric cancer; ADJ, adjacent to cancer; NC, non-cancerous; Up, up-regulated; Down, down-regulated; TS-miR, tumor suppressor miRNA; OncomiR, oncogenic miRNA. (-) Not differentially expressed. (^*^) Putative role in GC. (1) Wu et al., 2015; (2) Zhang et al., 2017b; (3) Deng et al., 2017; (4) Tang et al., 2016; (5) Zhang et al., 2017a; (6) Wang et al., 2017; (7) Shen et al., 2015; (8) Qu et al., 2017; (9) Yu et al., 2019; (10) Cong et al., 2013; (11) Chen et al., 2016; (12) Cao et al., 2016; (13) Zhou et al., 2015.

Comparing GC with ADJ, only one miRNA (*hsa-miR-196a-5p*) was differentially expressed in GC (Figure 1 and Table 1). *hsa-miR-196a-5p* was significantly up-regulated in GC (*P* = 0.029) but had a low sensitivity/specificity relation in the ROC curve analysis (AUC < 0.85). *hsa-miR-196a-5p* was also up-regulated in the GC vs. NC comparison (Table 1 and Supplementary Table S1). The difference in the number of miRNAs D. E. in GC vs. ADJ compared with the previous analysis corroborates to premise of that these two tissues have similar miRNA expression profiles.

## Discussion

miRNA deregulation is closely related to gastric cancer development (Wu et al., 2014; Zhang et al., 2014) and its relationship to field cancerization becomes evident (Assumpção et al., 2015; Pereira et al., 2019). In the search for new biomarkers and a better understanding of epigenetic field cancerization in GC, we evaluated the global expression profile of miRNAs in NC, ADJ, and GC gastric samples. Our data showed that all three types of tissue share many differentially expressed miRNAs and present miRNAs that occur exclusively in either ADJ or GC tissues, behaving as molecular signatures for those conditions (Tables 1, 2).

Among the deregulated miRNAs only in GC (Table 1), seven up-regulated (*hsa-miR-135b-5p*, *-miR-196a-5p*, *-miR-196b-5p*, *-miR-215-5p*, *-miR-224-5p*, -*miR-615-3p*, and *-miR-25-5p*) and five down-regulated (*hsa-miR-135a-5p*, *-miR-148a-3p*, *-miR-148a-5p*, *-miR-153-3p*, and *-miR-375*) miRNAs are likely oncomiRs and TS-miRs, respectively. In fact, studies demonstrate that *miR-135b* (Wang et al., 2012; Lu et al., 2018; Shao et al., 2019), *miR-196a* (Pan et al., 2017), *miR-196b* (Liao et al., 2012), *miR-215* (Deng et al., 2014), *miR-224* (He et al., 2017), *miR-615-3p* (Wang et al., 2018), and *miR-25* (Li B.S. et al., 2015; LArki et al., 2018) were reported as oncomiRs, while *miR-135a* (Zhang et al., 2016; Xie et al., 2019), *miR-148a* (Zheng et al., 2011), *miR-153* (Wang and Liu, 2016; Ouyang et al., 2018) and *miR-375* (Ding et al., 2010; Chen et al., 2017; Hwang et al., 2018) were reported as TS-miRs in GC. These results suggest that the joint deregulation of both oncomiRs and TS-miRs is required for the support and progression of GC.

*hsa-miR-196a-5p* was up-regulated in GC when comparing to ADJ and NC tissues; at the same time, it was not differentially expressed between the ADJ and NC tissues. Deregulation of this miRNA is important for GC progression because it promotes cell proliferation by down-regulating the expression of *CDKN1B* (p27^*kip*1^) tumor suppressor (Sun et al., 2012) and invasion and epithelial to mesenchymal transition of cancer stem cells by down-regulating the expression of *SMAD4* in GC (Pan et al., 2017). In addition, *hsa-miR-196a* has been associated with metastasis in lymph nodes and the clinical stage of GC (Li H.L. et al., 2015).

Nine miRNAs were found to be up-regulated only in ADJ tissue (*hsa-miR-let7-c-5p*, *-miR-99a-5p*, *-miR-100-5p*, *-miR-133a-3p*, *-miR-133b-3p*, *-miR-143-5p*, *-miR-145-3p*, *-miR-145-5p*, and *-miR-320b*) and may act as TS-miRs in this tissue. Studies have shown that *miR-let7-c* (Tsai et al., 2015), *miR-133a* (Qiu et al., 2014; Zhang et al., 2018), *miR-133b* (Qiu et al., 2014; Yang et al., 2017), *miR-143-5p* (Wu et al., 2013; Lei et al., 2017), *miR-145* (Qiu et al., 2014; Lei et al., 2017), *miR-100-5p* (Shi et al., 2015), and *miR-320b* (Zhao et al., 2017) are down-regulated in GC and play the role of TS-miRs because they inhibit proliferation, migration, invasion, and cell cycle progression.

Gene enrichment analysis in KEGG pathways revealed that these miRNAs regulate genes involved in important pathways that contribute to gastric carcinogenesis, such as the PI3K-Akt, HIF-1, Ras, Rap1, ErbB, and MAPK signaling pathways (Figures 5A,B). These pathways control important cell functions such as proliferation, migration, invasion, and progression of the cell cycle. The overexpression of TS-miRs in ADJ tissue may be a mechanism to compensate for preexisting molecular alterations in an attempt to contain the tumorigenesis process. However, we believe that during the progression of carcinogenesis in ADJ tissue, the up-regulated TS-miRs identified herein are inversely deregulated (become down-regulated), contributing to the eventual onset, establishment and progression of GC. In a previous study, we reported the overexpression of the TS-miR *hsa-miR-29c* in adjacent to the gastric cancer tissues and its down-regulation in GC and NC tissues (Pereira et al., 2019).

The intense inflammatory process to which the adjacent tissue is subjected may be one of the causes of over representation of these TS-miRs, since inflammation can alter the local microenvironment by stimulating the expression of some miRNAs. Studies have shown that IL-6 and IL-17 pro-inflammatory interleukins activity may stimulate the expression of miRNAs with known oncogenic activity (*miR-21* and *miR-135b*) in tissues submitted to the intense inflammatory process (Löffler et al., 2007; Iliopoulos et al., 2010; Matsuyama et al., 2011; Rozovski et al., 2013; Singh et al., 2015). Another possibility would be the existence of pre-cancerous lesions in adjacent tissues (e.g., atrophic and non-atrophic gastritis and intestinal metaplasia) and/or *Helicobacter pylori* infection, since the expression levels of many miRNAs (e.g., *miR-21*, *miR-29c*, *miR-135b*, *miR-155*, *miR-204*, and *miR-223*) may be change under these conditions (Link et al., 2015; Vidal et al., 2016; Pereira et al., 2019). Therefore, the local microenvironment and the histopathological characteristics of these tissues can directly influence the expression of many miRNAs, making them susceptible to eventual molecular alterations and carcinogenesis.

Our data (overexpression of nine TS-miRs only in adjacent to the gastric cancer tissues) corroborate with the findings of Aran et al. (2017), who used the transcriptomic profile of normal tissue adjacent to the tumor (NAT) to demonstrate that this tissue has an intermediate and unique expression profile when compared to truly normal and cancerous tissues. Therefore, despite the similarity between adjacent and tumor tissues, the former is not a malignant tissue, but it is not a molecularly normal tissue either (De Assumpção et al., 2016; Aran et al., 2017).

Many genetic and epigenetic changes identified in GC are shared by ADJ tissue (Assumpção et al., 2015; Takeshima et al., 2015; Aran et al., 2017; Vidal et al., 2017; Yoshida et al., 2017; Pereira et al., 2019). Probably some of these shared alterations were already present in the adjacent tissue before the onset of the tumor *in situ* and contributed to its establishment, making this tissue still susceptible to carcinogenesis even after surgical removal of the tumor.

We found that both ADJ and GC tissues share the up-regulation of *hsa-miR-125b-5p* and *hsa-miR-125b-1-3p* oncomiRs and the down-regulation of *hsa-miR-200a-3p* and *hsa-miR-873-5p* TS-miRs, suggesting that the joint deregulation of both oncomiRs and TS-miRs is required for the progression of gastric carcinogenesis. In addition, seven miRNAs (*hsa-miR-125b-5p*, *-miR-125b-1-3p*, *-miR-200a-3p*, *-miR-218-1-3p*, *-miR-490-3p*, *-miR-873-5p*, and *-miR-493-5p*) are deregulated in both ADJ and GC tissues when compared to NC tissue; however, these miRNAs are not D. E. between ADJ and GC tissues. Our results suggest that these two tissues share molecular alterations and that ADJ is an epigenetically altered tissue. Five miRNAs (*hsa-miR-125b-5p*, *-miR-125b-1-3p*, *-miR-493-5p*, *-miR-200a-3p*, and *-miR-873-5p*) can promote tumor onset and progression, as studies have shown that the deregulation of *miR-125b* (Wu et al., 2015; Zhang et al., 2017b), *miR-493-5p* (Zhou et al., 2015), *miR-200a* (Cong et al., 2013), and *miR-873* (Cao et al., 2016; Chen et al., 2016) contributes to cell proliferation, migration, invasion, and cell growth in GC.

Although we have not performed further experimental assays to confirm the deregulation and the functional role of the identified miRNAs in our study, the literature consistently supports and corroborates our findings and hypothesis through studies that have applied safe, sensitive and reliable techniques and methods (e.g., RT-qPCR, Western Blot, Cell and/or Reporter Assays) that provides strong evidences of these miRNAs’ deregulation in GC (Ding et al., 2010; Zheng et al., 2011; Liao et al., 2012; Sun et al., 2012; Wang et al., 2012, 2017, 2018; Cong et al., 2013; Wu et al., 2013, 2015; Deng et al., 2014, 2017; Qiu et al., 2014; Li B.S. et al., 2015; Shen et al., 2015; Shi et al., 2015; Tsai et al., 2015; Zhou et al., 2015, 2019; Zuo et al., 2015; Cao et al., 2016; Chen et al., 2016, 2017; Gao et al., 2016; Tang et al., 2016; Vidal et al., 2016; Wang and Liu, 2016; Zhang et al., 2016, 2017a, 2017b, 2018; He et al., 2017; Lei et al., 2017; Pan et al., 2017; Qu et al., 2017; Yang et al., 2017; Zhao et al., 2017; Hwang et al., 2018; LArki et al., 2018; Lu et al., 2018; Magalhães et al., 2018; Ouyang et al., 2018; Pereira et al., 2019; Shao et al., 2019; Xie et al., 2019; Yu et al., 2019).

For the evaluation of the studied miRNAs as potential biomarkers, we selected three different groups of markers with AUC > 85%. Among the D. E. miRNAs only in GC, six miRNAs (*hsa-miR-141-3p*, *-miR-148a-3p*, *-miR-148b-5p*, *-miR-153-3p*, *-miR-196a-5p*, and *-miR-375*) are potentially useful in identifying patients with advanced disease (Figure 3). Among the deregulated miRNAs only in ADJ tissue, five (*hsa-miR-99a-5p*, *-miR-100-5p*, *-miR-125b-5p*, *-miR-145-3p*, and *-miR-145-5p*) are potentially useful in identifying patients susceptible to tumor development or the early stages of gastric cancer (Figure 4). Two miRNAs (*hsa-miR-200a-3p* and *hsa-miR-873-5p*) are potentially useful in identifying both patients with established disease and patients susceptible to developing it (Figure 7). Thus, these miRNAs may be a potential diagnostic alternative for this type of cancer because the currently available biomarkers do not have a good sensitivity/specificity relationship, which makes it difficult to diagnose the disease early and to start curative treatment.

Studies that analyzed the data from miRNA sequencing in gastric cancer demonstrated their deregulation in this type of tumor (Assumpção et al., 2015; Darnet et al., 2015; Liu et al., 2015; Liang et al., 2017). Among the miRNAs found in these studies, *hsa-miR-99a*, *-miR-100*, *-miR-133a/b-3p*, *-miR-135b*, *-miR-141*, *-miR-143*, *-miR-145*, *-miR-148a*, *-miR-196a/b*, *-miR-200a*, *-miR-218-1*, *-miR-215*, and *-miR-490* were also observed in the present study. In addition to those miRNAs, we also found 16 novel differentially expressed miRNAs in the gastric field cancerization.

Liu et al. (2015) analyzed miRNome in gastric cancer downloaded from the TCGA; however, this database basically has samples from European and Asian populations. These authors identified 54 D. E. miRNAs (using the P*adj* < 0.05 and |log2 (*fold change*)| > 3) and two miRNAs (*hsa-miR-133a*/*b*) were the most D. E. Liang et al. (2017) also analyzed miRNomes in GC using samples from the same database (TCGA) and identified 43 D. E. miRNAs (using the FDR < 0.001 and | log2 (*fold change*)| > 1.5); of these, 5 miRNAs *(hsa-miR-30a*, *-miR-135b*, *-miR-133b*, *-miR-143*, and *-miR-145*) were associated with patient survival time. We emphasize that the “normal” or “healthy” tissues used in these two studies are of patients with the disease (adjacent to the gastric cancer tissues), since the TCGA does not have expression data of gastric tissues of patients without the history of GC. By using samples from the Brazilian population (that has genetic admixture), we identified 30 miRNAs differentially expressed when comparing patients with GC and individuals without the history of GC (we considered P*adj* < 0.05 and | log_2_(*fold change*)| > 2). In addition, we found that the *hsa-miR-196a-5p* was D. E. between the GC and ADJ tissues. Many D. E. miRNAs identified by Liu et al. (2015) and Liang et al. (2017) were also identified in this study; however, there are differences in the number of D. E. miRNAs found among these studies (mainly in GC and ADJ analysis), which may be a consequence of: (i) the statistical criteria used, (ii) the number of samples used, and/or (iii) the ethnical and genetic characteristics of the studied populations. Our data are important because we analyzed the miRNA sequencing of a larger number of samples from the Brazilian population. This population has a genetic contribution from different parental populations, such as the European, African, Asian, and Amerindian ones (Santos et al., 2010; Andrade et al., 2018). The strong genetic substructure and admixture (Santos et al., 2010) of our population may interfere in the expression profile of some genes (Pinto et al., 2015; Dluzen et al., 2016). Therefore, this study provides important and relevant information about the expression profile of miRNAs associated to gastric field cancerization in populations with genetic substructure and admixture, such as the Brazilian one.

Overall, our study was able to demonstrate that the tissue adjacent to gastric cancer shares some epigenetic changes (miRNAs deregulation) present in the tumor and also has unique and exclusive alterations; therefore, it should not be used as a healthy and/or normal tissue as a benchmark for gastric cancer. Thus, we recommend the use of gastric samples from patients with no history of GC as a control to exclude any biases that the adjacent tissue may provide in the miRNAs’ expression profile in GC.

In this study, we also observed that the tissues adjacent to GC have an over representation of microRNAs with known tumor suppressor activities, suggesting that these microRNAs may represent a barrier against tumorigenesis within these pre-cancerous tissues prior to the eventual formation of a tumor. The excellent performance of the studied miRNAs in identifying with good sensitivity and specificity, both early and advanced stages of the disease, make them potentially useful as biomarkers and therapeutic targets for GC.

## Ethics Statement

This study was carried out in accordance with the recommendations of the National Health Council’s Research Guidelines Involving Human Beings (Res. CNS 466/12), Research Ethics Committees and National Research Ethics Commission. The protocol was approved by the Ethics Committee of the Center of Oncology Research of the Federal University of Pará (Protocol No. 1.081.340). All subjects gave written informed consent in accordance with the Declaration of Helsinki.

## Author Contributions

AP and FM study design, formal analyses, interpretation of data, and drafting of manuscript. ÂRS, PA, and SS study concept and design and obtaining funding. PA, MA, and AC provided clinical materials. SD histopathological evaluation. TV-S, AV, PP, LM, and AMRS prepared nucleic acids, sequencing libraries, and performed the experiments. All authors reviewed the manuscript.

## Conflict of Interest Statement

The authors declare that the research was conducted in the absence of any commercial or financial relationships that could be construed as a potential conflict of interest.
