# Supplementary material for: miRNome Reveals New Insights Into the Molecular Biology of Field Cancerization in Gastric Cancer
Source: Front Genet. 2019 Jun 19;10:592. doi: 10.3389/fgene.2019.00592 (PMC6593062; doi:10.3389/fgene.2019.00592)
Supplement: Supplementary file 1 [file Image_1.pdf]

Supplementary Material

miRNome reveals new insights on molecular biology of the field cancerization in gastric cancer

Adenilson Pereira[1,#], Fabiano Moreira[1,2,#], Tatiana Vinasco-Sandoval[1], Adenard Cunha[2], Amanda Vidal[1], André Ribeiro-dos-Santos[1], Pablo Pinto[1], Leandro Magalhães[1], Mônica Assumpção[2], Samia Demachki[2], Sidney Santos[1,2], Paulo Assumpção[2], Ândrea Ribeiro-dos-Santos[1,2,*]

[1] Laboratory of Human and Medical Genetics, Institute of Biological Sciences, Federal University of Pará, Belém, PA, Brazil.
[2] Research Center on Oncology, Federal University of Pará, Belém, PA, Brazil.

[#]Authors contributed equally to this study.

* Correspondence: Dr. Ândrea Ribeiro-dos-Santos akelyufpa@gmail.com

Supplementary Figure S1

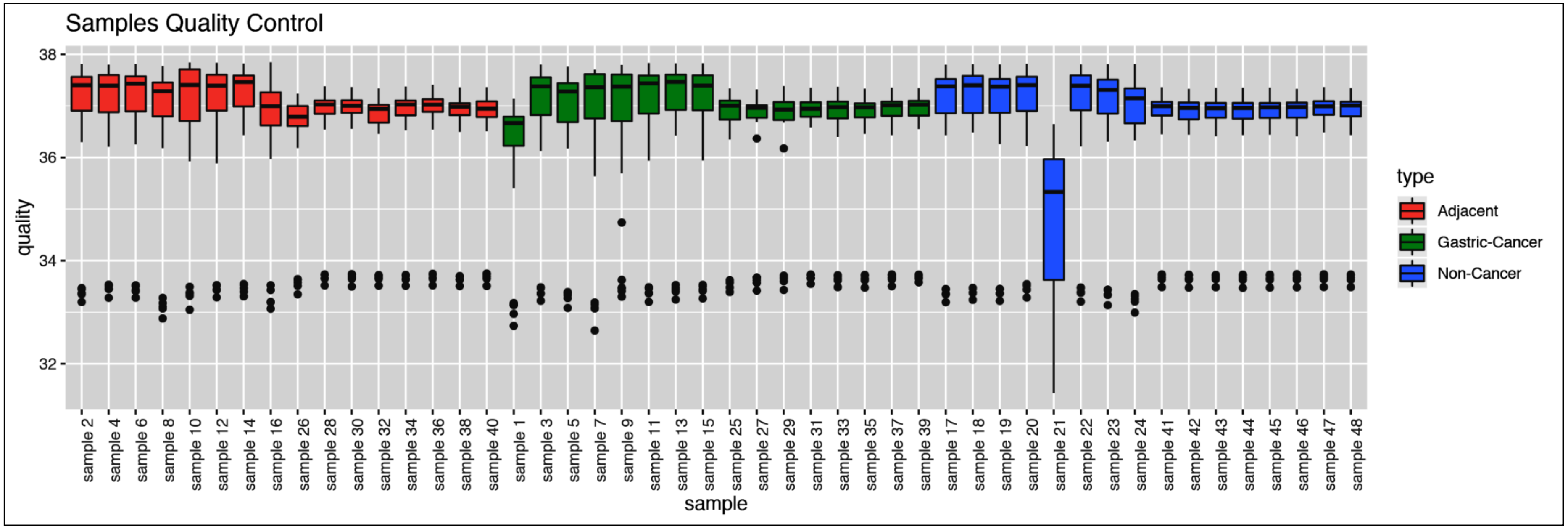

Figure S1: miRNAs reads quality control of the studied samples. Reads quality value (QV) in Phred scale of the forty eight sequenced samples after trimming and filtering by Trimmomatic tool (Bolger et al., 2014).

Reference:

Bolger, A.M., Lohse, M., Usadel, B. (2014) Trimmomatic: a flexible trimmer for Illumina sequence data**.** Bioinformatics. 30(15): 2114–2120.
