# Supplementary material for: miRNome Reveals New Insights Into the Molecular Biology of Field Cancerization in Gastric Cancer
Source: Front Genet. 2019 Jun 19;10:592. doi: 10.3389/fgene.2019.00592 (PMC6593062; doi:10.3389/fgene.2019.00592)
Supplement: Supplementary file 2 [file Table_1.DOC]

Supplementary Material

**miRNome reveals new insights on molecular biology of the field cancerization in gastric cancer**

Adenilson Pereira^1,#^, Fabiano Moreira^1,2,#^, Tatiana Vinasco-Sandoval^1^, Adenard Cunha^2^, Amanda Vidal^1^, André Ribeiro-dos-Santos^1^, Pablo Pinto^1^, Leandro Magalhães^1^, Mônica Assumpção^2^, Samia Demachki^2^, Sidney Santos^1,2^, Paulo Assumpção^2^, Ândrea Ribeiro-dos-Santos^1,2,*^

^1^ Laboratory of Human and Medical Genetics, Institute of Biological Sciences, Federal University of Pará, Belém, PA, Brazil.

^2^ Research Center on Oncology, Federal University of Pará, Belém, PA, Brazil.

^#^Authors contributed equally to this study.

* Correspondence: Dr. Ândrea Ribeiro-dos-Santos [akelyufpa@gmail.com](mailto:akelyufpa@gmail.com)

**Supplementary Table S1:** miRNAs significantly differentially expressed between the tissues GC *vs.* NC.

| **miRNA** | **|log_2_ *fold change*| > 2** | **Expression in GC** | **P-value*** |
| --- | --- | --- | --- |
| ***hsa-miR-125b-1-3p*** | 2,61 | Up | 2,00E-05 |
| ***hsa-miR-125b-5p*** | 2,00 | Up | 2,01E-06 |
| ***hsa-miR-135b-5p*** | 2,83 | Up | 1,85E-05 |
| ***hsa-miR-196a-5p*** | 5,30 | Up | 7,52E-10 |
| ***hsa-miR-196b-5p*** | 3,47 | Up | 3,92E-05 |
| ***hsa-miR-215-5p*** | 2,11 | Up | 1,91E-03 |
| ***hsa-miR-218-1-3p*** | 2,07 | Up | 3,57E-03 |
| ***hsa-miR-224-5p*** | 2,79 | Up | 4,18E-05 |
| ***hsa-miR-25-5p*** | 2,16 | Up | 7,59E-03 |
| ***hsa-miR-452-5p*** | 2,38 | Up | 5,10E-04 |
| ***hsa-miR-490-3p*** | 2,53 | Up | 6,17E-03 |
| ***hsa-miR-493-5p*** | 2,72 | Up | 8,67E-07 |
| ***hsa-miR-615-3p*** | 2,24 | Up | 2,10E-02 |
| ***hsa-miR-135a-5p*** | -2,10 | Down | 1,03E-04 |
| ***hsa-miR-141-3p*** | -3,03 | Down | 6,11E-11 |
| ***hsa-miR-148a-3p*** | -2,08 | Down | 3,68E-05 |
| ***hsa-miR-148a-5p*** | -2,21 | Down | 8,67E-07 |
| ***hsa-miR-153-3p*** | -2,45 | Down | 5,76E-08 |
| ***hsa-miR-200a-3p*** | -2,09 | Down | 1,38E-05 |
| ***hsa-miR-375*** | -2,58 | Down | 2,48E-06 |
| ***hsa-miR-873-5p*** | -3,06 | Down | 2,08E-05 |

GC: Gastric Cancer; ADJ: Adjacent; NC: Non-Cancer; Up: Uper-regulated; (*) P-*value* adjusted by FDR’s methods.
