# Supplementary material for: miRNome Reveals New Insights Into the Molecular Biology of Field Cancerization in Gastric Cancer
Source: Front Genet. 2019 Jun 19;10:592. doi: 10.3389/fgene.2019.00592 (PMC6593062; doi:10.3389/fgene.2019.00592)
Supplement: Supplementary file 3 [file Table_2.DOC]

Supplementary Material

**miRNome reveals new insights on molecular biology of the field cancerization in gastric cancer**

Adenilson Pereira^1,#^, Fabiano Moreira^1,2,#^, Tatiana Vinasco-Sandoval^1^, Adenard Cunha^2^, Amanda Vidal^1^, André Ribeiro-dos-Santos^1^, Pablo Pinto^1^, Leandro Magalhães^1^, Mônica Assumpção^2^, Samia Demachki^2^, Sidney Santos^1,2^, Paulo Assumpção^2^, Ândrea Ribeiro-dos-Santos^1,2,*^

^1^ Laboratory of Human and Medical Genetics, Institute of Biological Sciences, Federal University of Pará, Belém, PA, Brazil.

^2^ Research Center on Oncology, Federal University of Pará, Belém, PA, Brazil.

^#^Authors contributed equally to this study.

* Correspondence: Dr. Ândrea Ribeiro-dos-Santos [akelyufpa@gmail.com](mailto:akelyufpa@gmail.com)

**Supplementary Table S2:** miRNAs significantly differentially expressed between the tissues ADJ *vs.* NC.

| **miRNA** | **|log_2_ *fold change*| > 2** | **Expression in ADJ** | **P-value*** |
| --- | --- | --- | --- |
| ***hsa-let-7c-5p*** | 2,17 | Up | 1,06E-05 |
| ***hsa-miR-100-5p*** | 2,08 | Up | 2,62E-06 |
| ***hsa-miR-125b-1-3p*** | 2,85 | Up | 1,41E-04 |
| ***hsa-miR-125b-5p*** | 2,39 | Up | 2,39E-08 |
| ***hsa-miR-133a-3p*** | 2,81 | Up | 8,17E-07 |
| ***hsa-miR-133b*** | 2,67 | Up | 1,43E-03 |
| ***hsa-miR-143-5p*** | 2,16 | Up | 3,20E-04 |
| ***hsa-miR-145-3p*** | 2,32 | Up | 6,06E-08 |
| ***hsa-miR-145-5p*** | 2,46 | Up | 1,76E-11 |
| ***hsa-miR-218-1-3p*** | 2,14 | Up | 1,09E-02 |
| ***hsa-miR-320b*** | 2,24 | Up | 2,06E-03 |
| ***hsa-miR-490-3p*** | 3,80 | Up | 4,65E-06 |
| ***hsa-miR-493-5p*** | 2,18 | Up | 5,96E-04 |
| ***hsa-miR-99a-5p*** | 2,19 | Up | 1,31E-08 |
| ***hsa-miR-200a-3p*** | -2,11 | Down | 4,65E-06 |
| ***hsa-miR-873-5p*** | -2,07 | Down | 1,96E-03 |

(*) P-*value* adjusted by FDR’s methods.
