# Supplementary material for: miRNome Reveals New Insights Into the Molecular Biology of Field Cancerization in Gastric Cancer
Source: Front Genet. 2019 Jun 19;10:592. doi: 10.3389/fgene.2019.00592 (PMC6593062; doi:10.3389/fgene.2019.00592)
Supplement: Supplementary file 4 [file Table_3.DOC]

Supplementary Material

**miRNome reveals new insights on molecular biology of the field cancerization in gastric cancer**

Adenilson Pereira^1,#^, Fabiano Moreira^1,2,#^, Tatiana Vinasco-Sandoval^1^, Adenard Cunha^2^, Amanda Vidal^1^, André Ribeiro-dos-Santos^1^, Pablo Pinto^1^, Leandro Magalhães^1^, Mônica Assumpção^2^, Samia Demachki^2^, Sidney Santos^1,2^, Paulo Assumpção^2^, Ândrea Ribeiro-dos-Santos^1,2,*^

^1^ Laboratory of Human and Medical Genetics, Institute of Biological Sciences, Federal University of Pará, Belém, PA, Brazil.

^2^ Research Center on Oncology, Federal University of Pará, Belém, PA, Brazil.

^#^Authors contributed equally to this study.

* Correspondence: Dr. Ândrea Ribeiro-dos-Santos [akelyufpa@gmail.com](mailto:akelyufpa@gmail.com)

**Supplementary Table S3:** Functional annotation for validated target genes of TS-miRs differentially expressed in adjacent to gastric cancer *vs.* non-cancer samples, identified in this study.

| **KEGG Pathway** | **Genes** | **Fold Enrichment** | **P-*value**** |
| --- | --- | --- | --- |
| **PI3K-Akt signaling pathway** | *FGFR3, VEGFA, MYC, IGF1R, EGFR, BCL2L1, MTOR, NRAS* | 12.3 | 3.9E-05 |
| **Bladder cancer** | *FGFR3, VEGFA, MYC, EGFR, NRAS* | 64.8 | 2.2E-05 |
| **Pathways in cancer** | *FGFR3, VEGFA, MYC, IGF1R, EGFR, BCL2L1, MTOR, NRAS* | 10.8 | 3.2E-05 |
| **Central carbon metabolism in cancer** | *FGFR3, MYC, EGFR, MTOR, NRAS* | 41.5 | 6.7E-05 |
| **MicroRNAs in cancer** | *FGFR3, VEGFA, MYC, EGFR, HMGA2, MTOR, NRAS* | 13.0 | 6.0E-05 |
| **Proteoglycans in cancer** | *VEGFA, MYC, IGF1R, EGFR, MTOR, NRAS* | 15.9 | 1.8E-04 |
| **HIF-1 signaling pathway** | *SERPINE1, VEGFA, IGF1R, EGFR, MTOR* | 27.1 | 2.1E-04 |
| **Ras signaling pathway** | *FGFR3, VEGFA, IGF1R, EGFR, BCL2L1, NRAS* | 14.1 | 2.5E-04 |
| **Transcriptional misregulation in cancer^§^** | *MYC, IGF1R, EGFR, HMGA2, BCL2L1, SP1* | 15.8 | 1.3E-03 |
| **Glioma** | *FGFR3, EGFR, MTOR, NRAS* | 32.7 | 1.4E-03 |
| **Rap1 signaling pathway** | *FGFR3, VEGFA, IGF1R, EGFR, NRAS* | 12.6 | 2.6E-03 |
| **ErbB signaling pathway** | *MYC, EGFR, MTOR, NRAS* | 24.4 | 2.8E-03 |
| **Prostate cancer** | *IGF1R, EGFR, MTOR, NRAS* | 24.1 | 2.8E-03 |
| **Estrogen signaling pathway** | *FKBP5, EGFR, SP1, NRAS* | 21.4 | 3.5E-03 |
| **Choline metabolism in cancer** | *EGFR, SP1, MTOR, NRAS* | 21.0 | 3.5E-03 |
| **Signaling pathways regulating pluripotency of stem cells** | *FGFR3, IGF1R, EGFR, NRAS* | 15.1 | 8.3E-03 |
| **Endometrial cancer** | *MYC, EGFR, NRAS* | 30.6 | 1.7E-02 |
| **Acute myeloid leukemia** | *MYC, MTOR, NRAS* | 28.4 | 1.9E-02 |
| **Pancreatic cancer** | *VEGFA, EGFR, BCL2L1* | 24.5 | 2.4E-02 |
| **Melanoma** | *IGF1R, EGFR, NRAS* | 22.4 | 2.7E-02 |
| **Chronic myeloid leukemia** | *MYC, BCL2L1, NRAS* | 22.1 | 2.6E-02 |
| **MAPK signaling pathway** | *FGFR3, MYC, EGFR, NRAS* | 8.33 | 3.2E-02 |
| **GnRH signaling pathway** | *EGFR, MMP14, NRAS* | 17.5 | 3.8E-02 |

*P-*value* adjusted by Benjamin Hochberg’s correction.
